# Caffeine Induces G0/G1 Cell Cycle Arrest and Inhibits Migration through Integrin αv, β3, and FAK/Akt/c-Myc Signaling Pathway

**DOI:** 10.3390/molecules26247659

**Published:** 2021-12-17

**Authors:** Pichitchai Meisaprow, Nithikoon Aksorn, Chanida Vinayanuwattikun, Pithi Chanvorachote, Monruedee Sukprasansap

**Affiliations:** 1 Graduate Student in Master of Science Program in Nutrition, Faculty of Medicine Ramathibodi Hospital and Institute of Nutrition, Mahidol University, Bangkok 10400, Thailand; pichitchai.mei@student.mahidol.ac.th; 2 Department of Clinical Pathology, Faculty of Medicine Vajira Hospital, Navamindradhiraj University, Bangkok 10300, Thailand; nithikoon@nmu.ac.th; 3 Division of Medical Oncology, Department of Medicine, Faculty of Medicine, Chulalongkorn University, Bangkok 10330, Thailand; Chanida.Vi@chula.ac.th; 4 Cell-Based Drug and Health Product Development Research Unit, Faculty of Pharmaceutical Sciences, Chulalongkorn University, Bangkok 10330, Thailand; 5 Department of Pharmacology and Physiology, Faculty of Pharmaceutical Sciences, Chulalongkorn University, Bangkok 10330, Thailand; 6 Food Toxicology Unit, Institute of Nutrition, Mahidol University, Salaya Campus, Nakhon Pathom 73170, Thailand

**Keywords:** caffeine, lung cancer, metastasis, migration, cancer growth, c-Myc, integrin

## Abstract

Lung cancer is recognized as a major cause of mortality worldwide owing to its metastatic activity. Given the lack of solid information regarding the possible effects of caffeine, one of the most consumed natural psychoactive substances, on molecular signaling pathways implicated in the aggressive behavior of lung cancer, our study aimed to evaluate the effect and mechanism of caffeine on metastasis-related mechanisms. The results revealed that caffeine treatment at concentrations of 0–500 µM caused no direct cytotoxic effects on NCI-H23 cells. Treatment of cells with caffeine showed good potential to inhibit cell proliferation at 48 h and induced significant cell cycle arrest at the G0/G1 phase. Concerning metastasis, caffeine was shown to reduce filopodia formation, inhibit migration and invasion capability, and reduce the ability of cancer cells to survive and grow in an anchorage-independent manner. Moreover, caffeine could attenuate the formation of 3D tumor spheroids in cancer stem cell (CSC)-enriched populations. With regard to mechanisms, we found that caffeine significantly altered the integrin pattern of the treated cells and caused the downregulation of metastasis-associated integrins, namely, integrins αv and β3. Subsequently, the downstream signals, including protein signaling and transcription factors, namely, phosphorylated focal adhesion kinase (p-FAK), phosphorylated protein kinase B (p-Akt), cell division cycle 42 (Cdc42), and c-Myc, were significantly decreased in caffeine-exposed cells. Taken together, our novel data on caffeine-inhibiting mechanism in relation to metastasis in lung cancer could provide insights into the impact of caffeine intake on human diseases and conditions.

## 1. Introduction

Human lung cancer has been identified as one of the most critical types of cancers, accounting for nearly one in five of all cancer-related deaths worldwide [1]. Although various therapeutic strategies have been developed and improved in the past decade, the overall survival rate of patients with lung cancer is still low [2]. Most cancers are detected as metastatic cancers at the time of diagnosis, and metastasis is the main cause of death and treatment failure in lung cancer cases [3]. Thus, the prevention of cancer metastasis via inhibition of associated mechanisms may be a way to improve the survival and prognosis of lung cancer patients.

Cancer metastasis is an extraordinarily complex event associated with many interrelated biological processes [4]. Cell migration has been accepted as an essential initial event of cancer metastasis that allows cancer cells to separate from the primary tumor into adjacent tissue and enter the circulatory system [5]. Cellular protrusions accompanied by focal adhesion (FA) turnover are crucial to the process of cell migration. Upon response to stimulus, cells induce actin polymerization which leads to an extension of cell protrusions together with the creation of new cell–extracellular matrix (ECM) adhesions through FA formation at the leading edge of cells. These processes result in the generation of the mechanical force that helps cells to move forward. Subsequently, ECM–cell interactions at the trailing edge are dissociated by the disassembly of old FAs [6,7]. In FA formation, integrins are transmembrane receptors that play a critical role in mediating cell adhesion to the ECM [8]. Integrin–ECM engagement also stimulates intracellular signaling pathways controlling various fundamental activities of the cells, such as proliferation, differentiation, movement, and survival. Normally, integrins are formed as at least 24 heterodimers via pairing between 18 α-subunits and 8 β-subunits in humans [9]. Several lines of studies have found that the upregulation of some integrin subunits, such as α5, αv, β1, and β3, is related to highly aggressive behaviors and metastasis in cancer [10,11]. In lung cancer, the overexpression of integrin α5, β1, and β3 is correlated with a reduction in overall survival and disease-free survival in early-stage lung cancer patients [12]. Additionally, it has been reported that heterodimers of integrin α5β1 and αvβ3 could promote EGF receptor signaling and invasion of lung cancer [13].

Regarding mechanisms, integrin clustering, by responding to the attachment of cells to ECM molecules, can directly recruit and stimulate focal adhesion kinase (FAK) activity [14]. FAK is activated by auto-phosphorylation at Y397 and forms a complex with intracellular proteins, which results in the activation of various downstream effectors, including cell division cycle 42 (Cdc42) and protein kinase B (Akt) [15,16]. Cdc42 plays a major role in enhancing cell protrusions called filopodia which facilitate cell migration [17]. Unusual upregulation or activation of Akt, frequently observed in many types of cancer, is associated with several necessary processes for cancer metastasis, e.g., escape from anoikis, migration, and proliferation [18]. One of the critical events in the Akt signaling pathway is the triggering of the functional stability of oncogenic transcription factor c-Myc [19]. In lung cancer, the expression of c-Myc is frequently dysregulated [20]. c-Myc overexpression in cancer is the cause of aggressive behaviors, including cell cycle progression apoptotic resistance and metabolic modulation. Moreover, recent evidence revealed that c-Myc is also implicated in the properties of cancer stem cells (CSC) [21,22].

Caffeine (1,3,7-trimethylxanthine) is one of the most important and ubiquitous purine alkaloids, found in several edible plants, such as coffee beans, cocoa beans, kola nuts, and tea leaves [23]. Previous studies reported that caffeine has various health benefits via physiological and biological effects, including antioxidant, anti-inflammatory, and antibacterial activities, as well as promoting lipid metabolism and regulating smooth muscle function [24,25]. Interestingly, caffeine has exhibited an anticancer activity by which it can attenuate cell proliferation, invasion, cell cycle progression, and induce apoptosis [26,27,28]. In addition, it has been reported that a combination of caffeine and certain chemotherapy drugs also enhance anticancer activity [29]. However, the potential effect of caffeine on the regulatory mechanism of lung cancer metastasis is still unclear and evidence is limited. Therefore, to better clarify and understand the influence of caffeine on suppressing metastasis-related behavior in lung cancer cells, we aimed to investigate the effect of caffeine on cell proliferation, migration, and the CSC-like phenotype, and the underlying mechanisms in a human lung cancer cell line (NCI-H23).

## 2. Results

### 2.1. The Effect of Caffeine on Cell Viability in Human Lung Cancer NCI-H23 Cells

**Figure 1:**
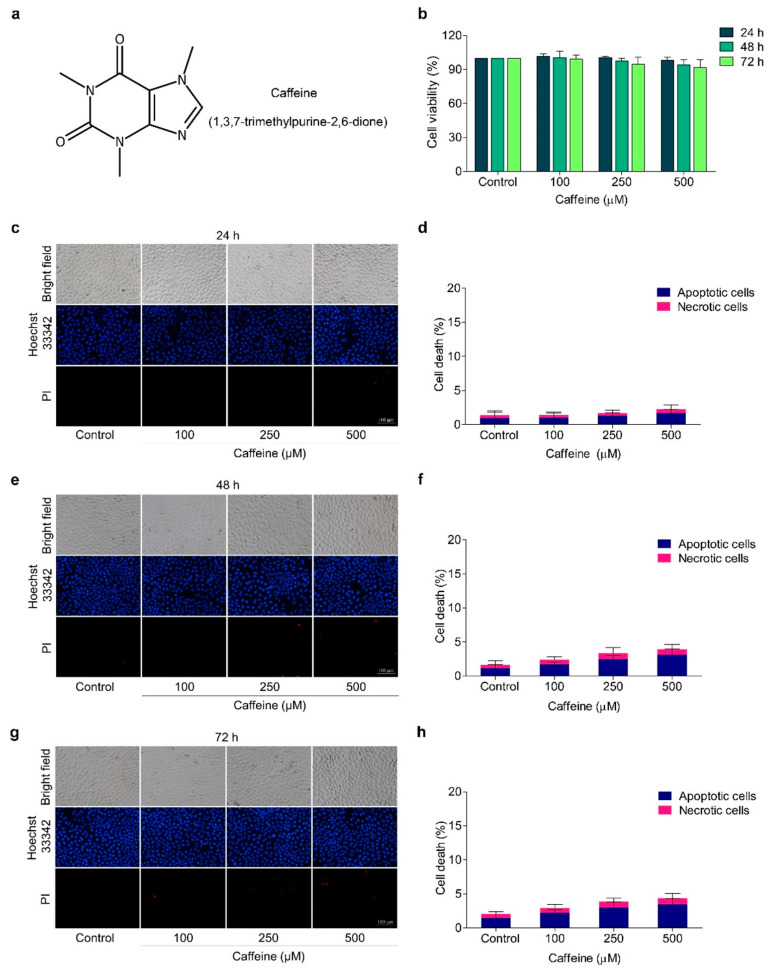
Effect of caffeine on viability of NCI-H23 cells. (**a**) Chemical structure of caffeine. (**b**) The cells were treated with caffeine at various concentrations (0–500 µM) for 24–72 h and cell viability was investigated with MTT assay. (**c**–**h**) The cells were treated with 0–500 µM of caffeine for 24–72 h and apoptotic and necrotic cells were examined by co-staining with Hoechst 33342 and PI. Images were visualized by fluorescence microscopy. Data are shown as mean ± SD (*n* = 3).

To assess the effects of caffeine on the metastasis regulatory mechanism of human lung cancer cells, we first investigated non-toxic concentrations of caffeine. The cells were treated with caffeine at various concentrations (0–500 µM) for 24, 48, and 72 h, and cell viability was evaluated by MTT assay. Results indicated that treatment of the cells with caffeine in all concentrations at the indicated times did not significantly alter the viability of the cells compared with the untreated control cells (Figure 1b). These results indicated that caffeine was relatively non-cytotoxic at the examined doses in NCI-H23 cells.

The hallmarks of apoptotic cells, such as condensed or fragmented nuclei, were monitored by blue fluorescence with Hoechst 33342 staining, while necrotic cells were indicated by red fluorescence with propidium iodide (PI) staining. Using double staining with Hoechst 33342 and PI, we confirmed that caffeine at the indicated doses and times had neither apoptosis- nor necrosis-inducing effects on the cells (Figure 1c–h). Based on these data, non-toxic concentrations (100–500 µM) of caffeine were therefore selected for further experiments on the inhibition of metastasis-related behavior in cancer cells.

### 2.2. Caffeine Attenuates Cell Proliferation and Cell Cycle Progression

**Figure 2:**
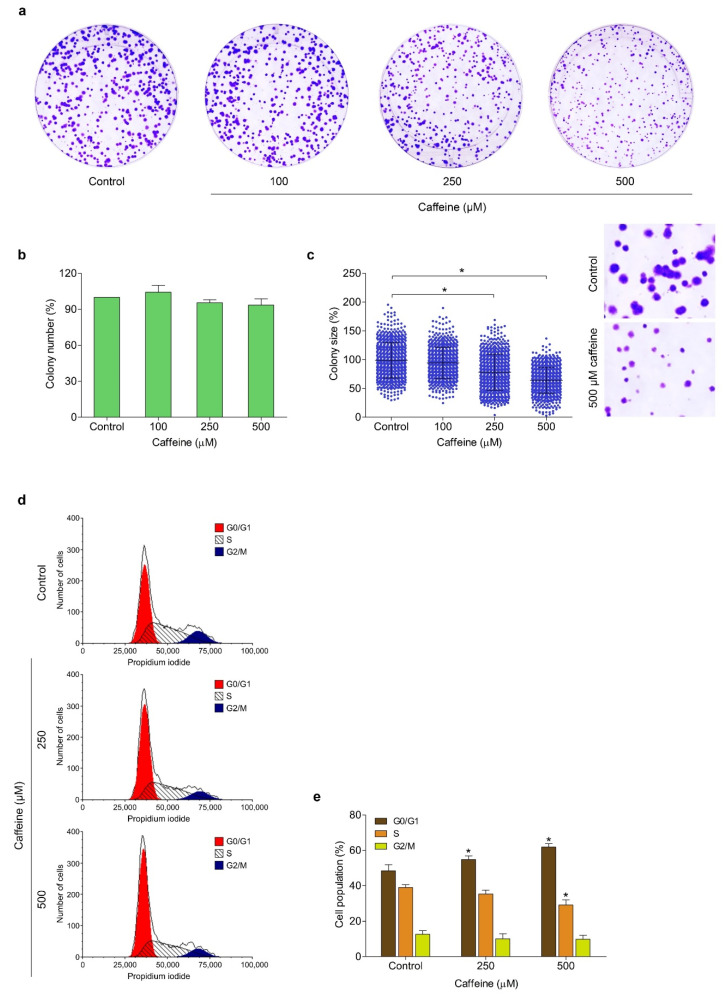
Effect of caffeine on cell proliferation and cell cycle in NCI-H23 cells. (**a**) The cells were treated with different concentrations of caffeine (0–500 µM) and incubated for 7 days to form colonies. The cell colonies were stained with crystal violet and visualized with a flatbed scanner. (**b**) The histogram shows the percentage of colony number. (**c**) The dot plot represents the percentage of colony size. (**d**,**e**) The cells were cultured with caffeine for 24 h and stained with propidium iodide solution. The cell cycle phase was analyzed by flow cytometry and results were presented as the percentage of the cell population in G1, S, and G2/M phases of the cell cycle. Data are presented as mean ± SD (*n* = 3). * *p* < 0.05 compared with the non-treated control.

The effect of caffeine on the growth and division of human lung cancer NCI-H23 cells was evaluated using colony formation assay. The cells were cultured in the presence of non-toxic concentrations of caffeine (100–500 µM) and allowed to form colonies for 7 days. Figure 2a shows the representative images of the colony formation assay. The results revealed that all concentrations of caffeine treatment did not cause a change in the number of generated colonies compared with the untreated control group (Figure 2b). However, the treatment with caffeine at 250 and 500 µM significantly reduced the size of colonies by 78.1% and 63.9%, respectively (Figure 2c). These results indicated that this compound could inhibit the proliferation of lung cancer cells.

Next, we investigated whether caffeine affects cell cycle progression. The cells were treated with caffeine at 0–500 µM for 48 h and the cell cycle profile was analyzed by PI staining and flow cytometry. As shown in Figure 2d,e, the proportions of cells in G0/G1 phase gradually increased from 48.44% (the untreated control group) to 54.85% and 60.87% with 250 and 500 µM caffeine, respectively. Meanwhile, the proportion of cells in S phase significantly decreased by 30.08% with 500 µM caffeine. Taken together, our results demonstrated that the caffeine treatment considerably induced G0/G1 phase arrest and reduced the S phase of the cell cycle, leading to inhibition of the proliferation of human lung cancer NCI-H23 cells.

### 2.3. Caffeine Suppresses Migration, Invasion, and Filopodia Formation

**Figure 3:**
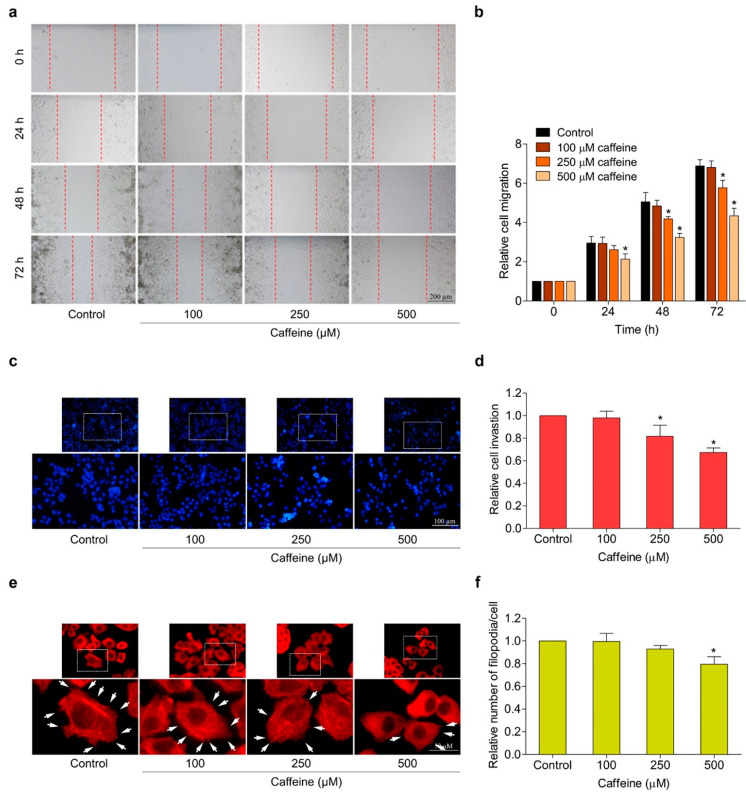
Effect of caffeine on cell migration, invasion, and filopodia formation in NCI-H23 cells. (**a**) Cell migration activity was assessed by wound-healing assay. The cells were treated with non-toxic doses of caffeine, and the movement of cells into the wound space was evaluated at 0, 24, 48, and 72 h. (**b**) The cell migration rate was represented as a relative value. (**c**) Cell invasion was determined with the transwell Boyden chamber. After incubating with caffeine for 24 h, the invaded cells were stained with Hoechst 33342 and determined by fluorescence microscopy. (**d**) Relative cell invasion was calculated from the number of invaded cells in the treatment groups divided by the control group. (**e**) The cells were cultured with caffeine for 24 h before staining with phalloidin–rhodamine. Filopodia formation was visualized by fluorescent microscopy and is indicated by white arrows. (**f**) The number of filopodia per cell was calculated as a relative value. Data are presented as mean ± SD (*n* = 3). * *p* < 0.05 compared with the non-treated control.

The migration and invasion of cancer cells are essential processes in metastasis. In this context, we investigated the potential effect of caffeine on the migration and invasion properties of human lung cancer NCI-H23 cells. The wound-healing assay was performed to evaluate the ability of cells to migrate. The cells were treated with caffeine at different concentrations (0–500 µM) for 24, 48, and 72 h and the migratory activity was investigated as described in the Section 4. Results showed that caffeine at the concentrations of 250 and 500 µM at 48 and 72 h significantly inhibited cell migration into the wound space, whereas 500 µM of caffeine treatment at 24 h could only suppress cell migration in NCI-H23 cells (Figure 3a,b). In addition, cell invasion was assessed by the transwell Boyden chamber coated with matrigel. The cells were cultured on a matrigel surface in the absence or presence of caffeine (0–500 µM) for 24 h. Our results showed that caffeine treatment at concentrations of 250 and 500 µM significantly suppressed the invasion of NCI-H23 cells through the matrigel layer in a dose-dependent manner (Figure 3c,d).

The formation of filopodia or cell protrusion is important for many crucial cellular functions, especially facilitating the movement of cancer cells. It was also used as a marker for verifying cell migration and invasion [30]. Therefore, we observed filopodia formation in NCI-H23 cells in response to caffeine treatment. The cells were cultured with various concentrations of caffeine (0–500 µM) for 24 h and subjected to the phalloidin–rhodamine staining assay as described in Materials and Methods. Figure 3e,f shows that caffeine was able to reduce the number of filopodia per cell. These findings demonstrate the anti-migration and anti-invasion capabilities of caffeine in human lung cancer NCI-H23 cells.

### 2.4. Caffeine Reduces Anchorage-Independent Growth and the CSC-like Phenotype of Hunam Lung Cancer NCI-H23 Cells

**Figure 4:**
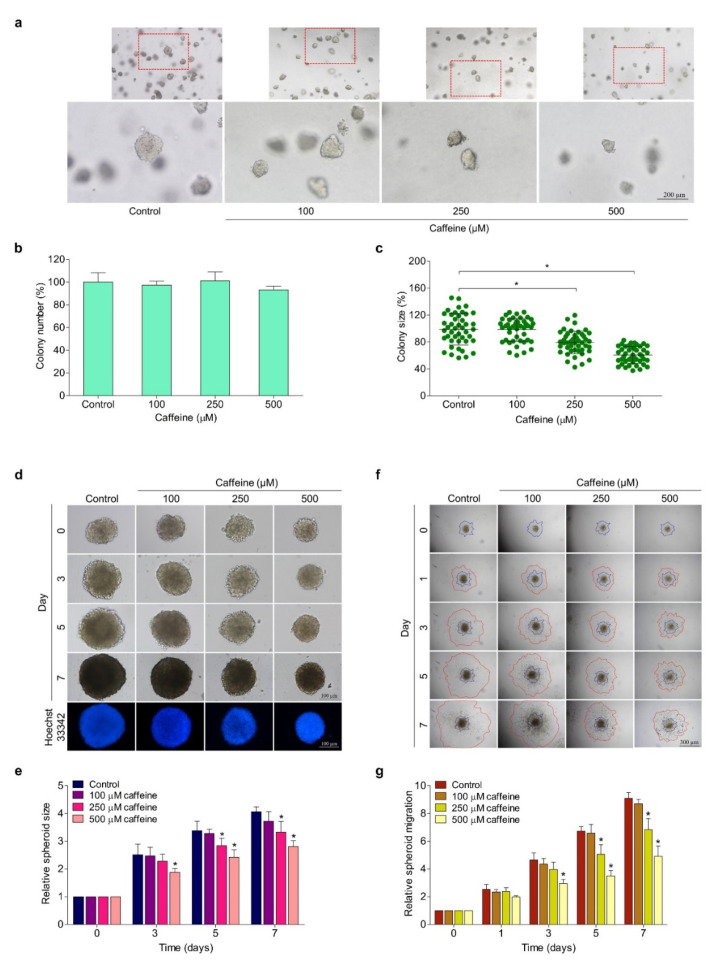
Effect of caffeine on anchorage-independent growth and CSC-like phenotypes of NCI-H23 cells. (**a**) The growth of cells in anchorage-independent conditions was investigated with soft agar colony-formation assay. The cell was cultured in soft agar containing various concentrations of caffeine (0–500 µM) for 7 days, then cell colonies were captured under an inverted microscope (**b**) Histogram shows the percentage of colony number. (**c**) The dot plot represents the percentage of colony size. (**d**,**e**) The CSC-like phenotypes were examined using a single 3D spheroid-formation assay. The secondary CSC-enriched spheroids were treated with 0–500 µM of caffeine, and the enlargement of spheroids was investigated at 0, 3, and 7 days. (**f**) The secondary spheroids were plated onto matrigel and treated with caffeine at concentrations of 0–500 µM. Cell migration away from the spheroid was imaged with an inverted microscope at 0, 3, 5, and 7 days. (**g**) The relative spheroid migration value was calculated from areas between the blue and red lines. Data are expressed as mean ± SD (*n* = 3). * *p* < 0.05 compared with the non-treated control.

The survival and growth of cancer cells in detached conditions or termed anchorage-independent growth have been related to anoikis resistance and the metastatic potential of cancer cells [31]. Cells were cultured for 7 days in soft agar in the absence or presence of caffeine (0–500 µM). The number and size of the cell colonies were examined by an inverted microscope. The colonies are shown in Figure 4a. We found that the treatment of cells with caffeine at concentrations of 250 and 500 µM inhibited the enlargement of the colonies’ size by about 21% and 34%, respectively, compared with untreated cells, whereas the reduction of the colonies’ numbers was not caused by caffeine. The results demonstrate that caffeine treatment (250–500 µM) significantly suppressed the growth of the cell colony size but did not reduce the ability of cancer cells to form colonies when compared with the untreated control (Figure 4b,c).

The formation of cancer spheroids under detachment conditions and the deprivation of growth factors are widely used to evaluate CSC phenotypes. CSC has a self-renewal ability and is involved in cancer metastasis [32]. Here, we examined the effect of caffeine on the CSC-like phenotype in NCI-H23 cells. The cells were subjected to a single 3D spheroid-formation assay. Figure 4d shows representative images of the size of CSC-rich spheroids treated with caffeine for 0, 3, 5, and 7 days. Treatment with the indicated concentrations of caffeine obviously suppressed the growth of CSC-rich spheroids in a time- and dose-dependent manner (Figure 4e). Our study found that a significant reduction in the size of NCI-H23 spheroids was first identified after treatment with caffeine for 3 days only at the highest concentration (500 µM), by which time the size reduction accounted for about 25% compared with the control group. Moreover, caffeine treatment for 7 days could distinctly reduce the enlargement of CSC-rich spheroids in both concentrations of 250 and 500 µM, which accounted for approximately 18% and 36%, respectively, compared with the control group (Figure 4e). These data indicate the inhibitory effect of caffeine on CSC-like phenotypes in lung cancer NCI-H23 cells in terms of both anchorage-independent growth and spheroid formation.

Next, we applied a spheroid-based migration assay to simulate the activity of tumor cell movement from a solid microtumor through obstacles to surrounding tissue based on interactions between cell–cell adhesions with extracellular matrix proteins [33]. The secondary tumor spheroids were cultured in matrigel pre-coated flat-bottom plates overnight. Afterwards, the single spheroids were treated with caffeine at concentrations of 0–500 µM and further cultured for 7 days. The representative images of the dissemination of the tumor cells on matrigel at 0, 1, 3, 5, and 7 days are shown in Figure 4f. The results found that in the untreated control group, the ability of the tumor cells to migrate on matrix proteins away from spheroids was rapid compared with the treatment group. Meanwhile, the treatments with caffeine were able to significantly reduce the migration of the tumor cells in a time- and dose-dependent manner (Figure 4f), although tumor spheroids treated with caffeine for 1 day did not affect the migration of tumor cells. The movement of cells away from the tumor spheroid was obviously suppressed after treatment with caffeine for 3 days at the highest concentration (500 µM) and for 5 and 7 days at concentrations of 250 and 500 µM (Figure 4g). Thus, caffeine could attenuate tumor cell migration in the context of the solid tumor microenvironment. Taken together, caffeine has an anti-metastatic potential with respect to the CSC-like phenotypes of NCI-H23 cells by which it can suppress growth under anchorage-independent conditions, as well as cancer migration and invasion.

### 2.5. Caffeine Suppresses Metastasis-Related Signaling Pathways via Integrin Alteration

**Figure 5:**
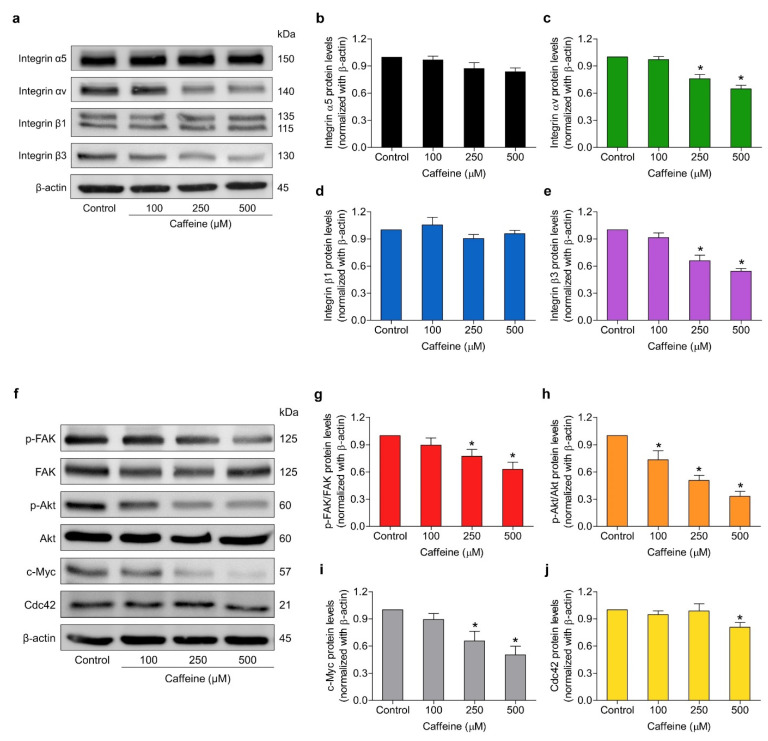
Effect of caffeine on the expression of metastasis-related proteins in NCI-H23 cells. (**a**,**f**) The cells were cultured with caffeine at concentrations of 0–500 µM for 24 h. The protein expression levels of integrins α5, αv, β1, β3, phosphorylated FAK (Tyr397), total FAK, phosphorylated Akt (Ser473), total Akt, c-Myc, and Cdc42 were investigated by western blot analysis (**b**–**e**,**g**–**j**). The relative protein levels were quantified by densitometry and normalized with β-actin to confirm the equal loading of samples. Data are presented as mean ± SD of at least three independent samples. * *p* < 0.05 compared with the non-treated control.

Integrins play essential roles in regulating cell adhesion to ECM proteins and activating intracellular signaling pathways necessary for many cellular processes, including cell survival, proliferation, and migration [9]. These cellular functions have been implicated in the highly aggressive properties of cancer cells which lead to the spread of cancer. To clarify the underlying mechanisms of caffeine on inhibiting the metastasis-related behaviors of lung cancer cells as described in the above findings, we investigated whether caffeine might alter the expression pattern of metastasis-involved integrins. The cells were treated with caffeine at concentrations of 0–500 µM for 24 h and the protein expression levels of integrin α5, αv, β1, and β3 were determined using western blot analysis. As shown in Figure 5a–e, the treated cells with the indicated concentrations of caffeine significantly attenuated the expression levels of integrin αv and β3 in a dose-dependent manner. Meanwhile, we found that the expression levels of integrin α5 and β1 were not significantly altered when compared with the control group. Furthermore, we examined the expression of the downstream signaling targets of integrins, including total FAK, the active form of FAK (phosphorylated at Try397; p-FAK), total Akt, the active form of Akt (phosphorylated at Ser473; p-Akt), and Cdc42, as well as c-Myc. These proteins have been clearly confirmed to make substantial contributions in regulating cancer cell migration, invasion, proliferation, and CSC phenotypes. The results demonstrated that caffeine treatment significantly reduced the expression of p-FAK and p-Akt proteins in a dose-dependent manner (Figure 5g,h), which correlated with the decrease in integrin αv and β3 levels. Likewise, the expressions of their downstream effectors, such as c-Myc and Cdc42 proteins, were downregulated in response to treatment with caffeine, as shown in Figure 5i,j.

**Figure 6:**
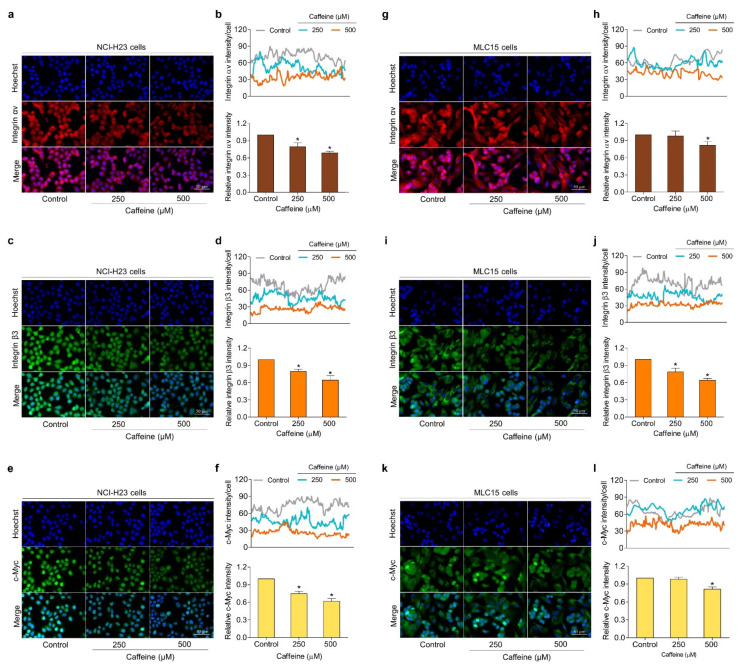
Effect of caffeine on the expression of metastasis regulatory proteins in both human lung cancer cells (NCI-H23 cells) and primary human lung cancer cells (MLC15 cells). The immunofluorescence assay was performed for analysis. (**a**–**f**) NCI-H23 cells or (**g**–**l**) MLC15 cells were treated with caffeine at concentrations of 0–500 µM for 24 h, then incubated with primary antibodies (integrin αv, integrin β3, and c-Myc) followed by co-staining with Alexa Fluor 594-labeled secondary antibody (for integrin αv; red) or Alexa Fluor 488-labeled secondary antibody (for integrin β3 and c-Myc; green) and Hoechst 33342. Confocal images were visualized under fluorescence microscopy and the fluorescence intensity was measured with ImageJ software. The histogram represents the relative intensity value of the cells. Data are expressed as mean ± SD of at least three independent experiments. * *p* < 0.05 compared with the non-treated control.

To confirm the inhibitory effect of caffeine on the expressions of integrin αv, β3, and c-Myc proteins, the immunofluorescence experiment was used to further evaluate both human primary lung cancer MLC15 cells and human lung cancer NCI-H23 cells. The cells were exposed to caffeine at concentrations of 0–500 µM for 24 h and subject to immunofluorescence staining. Our results found that the treatment of NCI-H23 cells with caffeine significantly decreased the expression levels of integrin αv, β3, and c-Myc in a dose-dependent manner (Figure 6a–f). For MLC15 cells, the integrin αv and c-Myc protein signals were slightly reduced, while integrin β3 was strongly diminished by the treatment with caffeine when compared with the untreated group (Figure 6g–l). Overall, these results suggest that caffeine inhibits the metastasis regulatory mechanism of lung cancer cells through integrins/Akt/c-Myc signaling.

## 3. Discussion

For many years lung cancer has been known as one of the most aggressive malignancies and is the main cause of cancer-associated fatalities in the world [1]. Epidemiological studies currently reveal that the five-year survival rate among lung cancer patients diagnosed between 2010 and 2016 for all stages combined is about 21% [3]. Significantly, survival estimates are often low when the cancer is first diagnosed, as more than half of all lung cancer cases are in the metastatic stage at diagnosis [3]. Therefore, the development of strategies to prevent cancer metastasis is of great importance. In fact, cancer metastasis is a rather complicated event related to several biochemical processes which lead to the dissemination of malignancy cells from their original parts and the successful colonization of different parts of the body [34]. For metastasis to be accomplished, cancer cells need to gain the capability to migrate and invade [35].

Recently, researchers have attempted to study the property of bioactive compounds derived from edible plants to suppress migration, invasion, and metastasis in many cancers [36,37]. The class of xanthine alkaloids, namely, caffeine, is a naturally occurring psychoactive substance that is consumed worldwide [23]. Various pieces of evidence have demonstrated that caffeine not only plays a role in stimulating the central nervous system but also has an anticancer effect. Cheng and colleagues found that treatment with caffeine could inhibit the progression of human glioblastoma by suppressing invasion and growth through cathepsin B and MAPK signaling [38]. Likewise, caffeine was reported to have the ability to promote an anti-tumor immune response via blockage of the adenosine pathway [39]. Furthermore, it was revealed that caffeine could induce atorvastatin to inhibit migration, invasion, and the formation of tumorspheres, and enhance apoptosis of prostate cancer cells [40]. In the present study, we therefore investigated the potential effect and underlying mechanism of caffeine on human lung cancer cells. Our result found that caffeine at non-toxic concentrations could notably suppress lung cancer NCI-H23 cell proliferation in a dose-dependent manner (Figure 2a–c). Consequently, we examined the preliminary mechanism involved in regulating cell proliferation, the ratio of cells in each cell cycle phase, and the expression of c-Myc in response to caffeine treatment. Our results revealed that caffeine could inhibit cell cycle progression by inducing G0/G1 phase arrest and decreasing S phase in tandem with depleted c-Myc levels (Figure 2d,e and Figure 5f,i). The proto-oncoprotein c-Myc acts as a nuclear transcription factor that regulates the encoding of many target genes associated with biological functions in normal and cancerous cells [41]. Deregulated overexpression of c-Myc has been widely discovered in numerous cancer types. It contributes to almost every step of carcinogenesis and tumor progression, including the relentless proliferation of cells, apoptosis resistance, angiogenesis, and the alteration of cellular metabolism [42]. In terms of cell proliferation, c-Myc has the key abilities to control cell cycle progression by promoting the transcription of its downstream genes for the cell cycle transition from G0/G1 into S phase and antagonizing cell cycle inhibitor activity [43]. Consistent with our above findings, research has shown that the downregulation of c-Myc protein expression results in cell cycle arrest in the G0/G1 phase and inhibits the proliferation of different cancers [44,45]. Indeed, after synthesis, the c-Myc protein is quite unstable with a very short half-life and is rapidly degraded through the ubiquitin-mediated proteasome system. However, these processes are often destroyed in cancer cells due to the irregularity of various cellular mechanisms, one of which is the over-activation of the Akt pathway. The enhancement of phosphorylated Akt has been reported to play a crucial role in blocking the degradation and increased functional stabilization of c-Myc protein [46,47]. In addition, some evidence indicates that an increase of c-Myc expression is relevant to the activation of the Akt signaling pathway [48]. Our results demonstrated that caffeine also suppressed the expression of phosphorylated Akt and c-Myc proteins (Figure 5f,h,i).

Moreover, the migration, invasion, and growth in an anchorage-independent manner of human lung cancer NCI-H23 cells were remarkably attenuated by exposure to caffeine at non-toxic concentrations (Figure 3a–d and Figure 4a–c). Cell adhesions to ECM ligands are an important requirement for cell migration and invasion. Integrins also play a role in this context. Integrins are αβ heterodimers of transmembrane receptors functioning as cell adhesion molecules that link specific ECM molecules and the cytoskeleton via the FA complex [10]. Generally, the engagement of integrins in the ECM produces dynamic cell–ECM interactions and supports mediated molecular signaling essential to the regulation of various cellular functions, such as proliferation, invasion, and movement [49]. Additionally, the anchorage of the cell to the ECM mediated by integrin can activate signaling necessary for cell survival, while the loss of such adhesion brings about detachment-induced apoptosis, known as anoikis [50]. Resistance to anoikis is one of the hallmarks of malignant cells and has contributed to success in metastasis [51]. Current evidence has revealed that an altered pattern of integrin expression due to an increase in certain subunits and a decrease in others is involved in aggressive cancer behaviors, including migration, avoidance of anoikis, and metastasis. The expression of the integrin β3 subunit can induce the upregulation of MMP-2 and promote the invasive potential of breast cancer cells [52]. Growth in anchorage-independent conditions and metastasis in pancreatic cancer have been promoted with the presence of integrin αvβ3 [53]. Similarly, the activation of integrin αvβ3 and downstream pathways were reported to facilitate the migration of lung cancer cells and influence anoikis resistance in ovarian cancer cells [54,55]. In contrast, the suppression of integrin β3 and αv with antisense therapy resulted in inhibiting angiogenesis, growth, and inducing apoptosis in hepatocellular carcinomas [56]. Our study indicated that caffeine treatment significantly decreases the expression of integrin β3 and αv in both NCI-H23 and MLC15 cells (Figure 5a–e and Figure 6a–d,g–j).

Regarding the mechanism of action, it is commonly known that integrin-mediated cell adhesion causes the recruitment and functional activity of FAK. Phosphorylation of FAK has been reported as the main driver in cell survival and motility through allowing the activation of downstream effectors, such as Akt and Cdc42 [16,57]. The unnatural overexpression or activation of Akt has been associated with an increased ability to proliferate, survive, and migrate in many cancers [58]. Cdc42 is one of the migration regulatory proteins. It can be stimulated to expand membrane protrusions called filopodia at the edge of the migrating cells. Abundant filopodia have been considered as characteristic of metastatic cells [59,60]. In response to caffeine, the expression of p-FAK, p-Akt, and Cdc42, as well as filopodia formation, were markedly reduced, corresponding to the downregulation of integrins (Figure 3e–f and Figure 5f–h,j). Based on these findings, we suggest that the suppressive effect of caffeine on cell migration, invasion, and anchorage-independent growth may be associated with a decrease in the levels of integrin αv, β3, and their down-stream signaling pathway.

Previous data demonstrated that the heterogeneous cell populations inside malignant tumors appear to include a small group of rare cells called CSC, which exhibit various unique properties that drive carcinogenesis and tumorigenesis [61]. The self-renewal capacity, differentiation into diverse lineages of cell populations, and other biological features similar to normal stem cells are crucial characteristics of CSC [62]. Remarkably, CSC can be therapy-resistant, meaning that although successful treatments may destroy large groups of tumor cells, a residue may survive, resulting in cancer relapse and more invasiveness [63]. Additionally, evidence has been reported that CSC is the main factor responsible for cancer aggression, maintenance, avoidance of the immune system, and metastasis [64,65]. Single cancer cell culture under detachment conditions with the nutrients and growth factors needed to form tumor spheroids, known as single 3D spheroid-formation assay, has been used to evaluate the CSC property [66,67]. Our results distinctly revealed that the enlargement of NCI-H23 tumor spheroids in a CSC-enriched population was diminished with caffeine treatment (Figure 4d,e). According to the results, caffeine could reduce the expression of integrin αv and β3 protein (Figure 5a,c,e), which play a role in promoting the CSC phenotype; therefore, we used these proteins as markers of CSC. These results are consistent with Seguin and colleagues’ report that integrin αvβ3 is the key marker and driver of CSC properties and drug resistance in lung and pancreatic cancers [68]. In breast cancer, aggressive stem-like behavior and slug expression was regulated by increasing integrin αvβ3 expression [69]. Moreover, transcription factors and intracellular signaling molecules, such as c-Myc and p-Akt, were accepted as important regulators for the biological activities of CSCs in different cancers [70]. Several studies indicated that the of these proteins has been implicated in the inhibition of CSC-like properties in lung cancer [32,71]. Thus, this study shows that caffeine is involved in a mechanism capable of suppressing lung cancer CSC-like properties which may be related to the downregulation of integrin αv, integrin β3, p-Akt, and c-Myc.

## 4. Materials and Methods

### 4.1. Chemicals and Reagents

Caffeine (purity ≥ 99%, Figure 1a), bovine serum albumin (BSA), Hoechst 33342, propidium iodide (PI), triton X-100, 3-(4,5-dimethylthiazol-2-yl)-2,5-diphenyltetrazolium bromide (MTT), tween-20, and skimmed milk powder were purchased from Sigma-Aldrich Co. (St. Louis, MO, USA). Matrigel was obtained from Corning Inc. (Bedford, MA, USA). Roswell Park Memorial Institute 1640 medium (RPMI), phosphate buffered saline (PBS), fetal bovine serum (FBS), penicillin/streptomycin, L-glutamic acid, and trypsin–EDTA (0.25%) were purchased from Gibco (Gaithersburg, MA, USA). Rhodamine–phalloidin was purchased from Invitrogen (Carlsbad, CA, USA). Dimethyl sulfoxide (DMSO), paraformaldehyde, glycerol, as well as immobilon classico western HRP substrate, were obtained from Merck (Darmstadt, Hesse, Germany). Protease inhibitor cocktail were purchased from Thermo Fisher Scientific (Rockford, IL, USA). Agarose was obtained from Bio-Rad Laboratories (Hercules, CA, USA). Radioimmunoprecipitation assay (RIPA) buffer, anti-bodies against, integrin αv (#4600), integrin α5 (#4705), integrin β1 (#4706), integrin β3 (#4702), FAK (#3285), p-FAK (Tyr397; #3283), Akt (#9272), p-Akt (Ser473 #4060), c-Myc (#18583), Cdc42 (#2466), β-actin (#4970), and horseradish peroxidase-coupled secondary antibodies were purchased from Cell Signaling Technology (Danvers, MA, USA). Caffeine was primarily dissolved in sterile deionized water, then diluted with culture medium to achieve the desired concentrations.

### 4.2. Cell Cultures

Human lung cancer cell lines (NCI-H23) were obtained from American Type Culture Collection (ATCC, Manassas, VA, USA). Human primary lung cancer cells (MLC15) were collected from pleural effusions of patients who have been diagnosed with recurrent or advanced stage non-small cell lung cancer at King Chulalongkorn Memorial Hospital. Briefly, pleural effusion (500–1000 mL) obtained through thoracentesis was centrifuged at 1500 rpm for 10 min at 4 °C to isolate cancer cells. The protocol has been approved by the Ethics Committee of the Faculty of Medicine, Chulalongkorn University (IRB 365/62). NCI-H23 cells and MLC15 cells were cultured in RPMI medium containing 10% (*v/v*) FBS, 100 units/mL each of penicillin and streptomycin, and 2 mM L-glutamine. The cells were maintained in a humidified incubator at 37 °C with 5% CO_2_.

### 4.3. Cell Viability Assay

The cytotoxic effect of caffeine on NCI-H23 cells was investigated using an MTT assay. The cells were cultured in 96-well plates at a density of 1 × 10^4^ cells/well. After incubating overnight, the cells were treated with various concentrations of caffeine (0–500 µM) for 24, 48, and 72 h. Then, the supernatants were removed, and the treated cells were incubated with MTT solution (0.4 mg/mL) at 37 °C for 4 h. Next, the solution was replaced with DMSO to dissolve formazan crystals. The absorbance was measured at a wavelength of 570 nm by a microplate reader (Anthros, Durham, NC, USA). The percentage of cell viability was calculated from the absorbance of treated cells relative to the absorbance of untreated cells.

### 4.4. Differential Nuclear Staining Assay

Nuclei condensation and DNA fragmentation in apoptotic cells and necrotic cells were evaluated via nuclear dual staining with Hoechst 33342 and PI, respectively. Briefly, NCI-H23 cells were seeded at a density of 1 × 10^4^ cells/well onto 96-well plates overnight, then treated with caffeine at various concentrations (0–500 µM) for 24, 48, and 72 h. Next, the specific treatment cells were washed in PBS and co-stained with 10 µg/mL of Hoechst 33342 and 5 µg/mL of PI for 15 min at 37 °C in darkness. Immediately, the cells were visualized and scored with fluorescence microscopy (Nikon ECLIPSE Ts2, Tokyo, Japan). The percentages of apoptotic and necrotic cells in each condition were estimated from 5 random fields of view.

### 4.5. Colony Formation Assay

NCI-H23 cells were harvested and suspended in complete medium at a density of 3 × 10^2^ cells/mL. The single-cell suspensions were added onto 6-well plates at a quantity of 1.5 mL/well and incubated at 37 °C overnight for cell attachment. After incubation, the complete medium was removed and the cells were treated with various concentrations of caffeine (0–500 µM). After allowing formation for 7 days, the cell colonies were washed with PBS, fixed with 4% (*v/v*) paraformaldehyde for 15 min, followed by staining with 0.5% (*w/v*) crystal violet solution for 10 min at room temperature. The morphology of colonies was visualized using a flatbed scanner (HP Scanjet 200, Palo Alto, CA, USA) after washing with demineralized water. The number and size of cancer colonies were detected by OpenCFU software [72].

### 4.6. Cell Cycle Analysis

NCI-H23 cells were cultured into 6-well plates at a density of 8 × 10^4^ cells/well with complete medium containing 10% (*v/v*) FBS overnight. After removing complete medium, the cells were rinsed twice with PBS and incubated with serum-free medium for 24 h. Next, serum-free medium was replaced with complete medium with or without the addition of caffeine (0–500 µM) and the cells were further cultured for 48 h. The treated cells were collected and fixed with 4% (*v/v*) paraformaldehyde for 10 min. Then, fixed cells were suspended in staining solution (0.1% (*v/v*) Triton X-100, 100 µg/mL RNase, and 20 µg/mL PI in PBS) and incubated for 15 min at room temperature in darkness. The proportions of the cells in different cell cycle phases (G0/G1, S, and G2/M) were determined by flow cytometry (Guava EasyCyte, Austin, TX, USA) and data were analyzed with FCS Express 7 Software (De Novo Software, Pasadena, CA, USA).

### 4.7. Migration and Invasion Assays

Cell migration was examined through the wound-healing assay. NCI-H23 cells were seeded onto 96-well plates at a density of 8 × 10^3^ cells/well and incubated at 37 °C until the cells grew to approximately 95% confluence. Next, the cell monolayer was scratched with a P200 sterile micropipette tip to generate a wound. Cell debris was removed by rinsing twice with PBS. After treatment with caffeine (0–500 µM), the progress of cell migration into the wound space was documented by taking photographs under an inverted microscope (Olympus, Melville, NY, USA) at 0, 24, 48, and 72 h. The wound area was measured with ImageJ software (National Institutes of Health, Bethesda, MD, USA).

Cell invasion was investigated using the transwell Boyden chamber with polycarbonate membrane filters (8 µm pore size; BD Bioscience, Bedford, MA, USA). The membrane of the upper chamber was pre-coated with 0.5% (*v/v*) matrigel in serum-free medium and incubated overnight at 37 °C for the gel to set. The cells were resuspended (density of 4 × 10^4^ cells/mL) in serum-free medium containing various concentrations of caffeine (0–500 µM). Afterward, the cell suspension was added to the upper chamber, while culture medium supplement with 10% (*v/v*) was transferred to the lower chamber. After incubation at 37 °C for 24 h, non-invasive cells on pre-coated membranes were removed by cotton swabs, while invasive cells at the bottom of the pre-coated membrane were fixed with 4% (*v/v*) paraformaldehyde for 10 min at room temperature. Next, the fixed cells were stained with 10 µg/mL of Hoechst 33342 for 10 min. Lastly, stained cells were captured and determined under a fluorescence microscope (Nikon ECLIPSE Ts2, Tokyo, Japan).

### 4.8. Cell Morphology and Filopodia Characterization

Phalloidin–rhodamine staining assay was used to investigate cell morphology and filopodia formation. The cells were cultured in 96-well plates overnight and then treated with caffeine at different concentrations for 24 h. The treated cells were rinsed twice with PBS and fixed 4% (*v/v*) paraformaldehyde for 10 min at room temperature. Next, the cells were permeabilized with 0.1% (*v/v*) Triton X-100 for 5 min and blocked in 0.2% (*w/v*) BSA for 30 min. Then, the blocked cells were stained with phalloidin–rhodamine solution (1:100 ratio) in PBS for 15 min. After washing with PBS, the stained cells were covered with 50% (*v/v*) glycerol to prevent dehydration. Cell morphology and filopodia characterization were imaged under a fluorescence microscope (Nikon ECLIPSE Ts2, Tokyo, Japan) with a 40× objective lens. The relative numbers of filopodia were calculated from the average number of filopodia per cell of the caffeine-treated cells divided by the average number of filopodia per cell of untreated cells.

### 4.9. Anchorage-Independent Growth Assay

The growth of cells under anchorage-independent conditions was investigated using the soft agar colony formation assay. The base agar was freshly prepared by diluting 1% (*w/v*) molten agarose with serum-free medium (1:1 ratio), then the mixture was added onto the bottom of 24-well plates. The agar was allowed to solidify at room temperature for 45 min. The cells were resuspended in complete medium (8 × 10^3^ cells/mL) with or without the addition of caffeine (0–500 µM). Next, the upper cellular layer was prepared by mixing these cell suspensions with 0.6% (*w/v*) agarose (1:1 ratio). The upper layer was gently added over base agar and the cells were allowed to form colonies for 7 days at 37 °C. To prevent desiccation, the complete medium (with or without caffeine) was filled onto the upper layer every 2 days. The tumor cell colonies were captured by an inverted microscope (Olympus, Melville, NY, USA). The number and size of the colonies were analyzed by ImageJ software (National Institutes of Health, Bethesda, MD, USA).

### 4.10. Single Three-Dimensional (3D)Spheroid-Formation Assay

Single 3D spheroid-formation assay was performed as previously described with slight modification [32]. The cells were cultured in 6-well ultra-low attachment plates at a density of 2.5 × 10^3^ cells/well with culture medium containing 1% (*v/v*) FBS for 7 days to form primary spheroids. Next, the obtained primary spheroids were collected, centrifuged at 1000 rpm for 5 min, and resuspended in culture medium containing 1% (*v/v*) FBS into single cells. The primary spheroid derived-cells were cultured again on 6-well ultra-low attached plates and allowed to form secondary CSC-enriched spheroids for 7 days. The secondary spheroids were transferred to 96-well ultra-low attachment plates at one spheroid per well and treated with caffeine at various concentrations. The alteration of the spheroid was documented by taking images at 0, 3, 5, and 7 days under an inverted microscope (Nikon ECLIPSE Ts2, Tokyo, Japan) At day 7, the single spheroids were stained with 10 µg/mL of Hoechst 33342 for 15 min at 37 °C in the darkness. The size of spheroids was analyzed with ImageJ software (National Institutes of Health, Bethesda, MD, USA).

### 4.11. Tumor Spheroid-Based Migration Assay

To investigate the inhibitory effect on tumor cell migration, the cells were generated as multicellular secondary spheroids, following the same protocol as described above. Next, a 96-well plate was pre-coated with 1% (*v/v*) matrigel in serum-free medium and allowed to harden overnight at 37 °C. The prepared secondary spheroids were transferred onto a matrigel pre-coated plate with a micropipette tip at one spheroid per well. After culturing overnight, the tumor spheroids were treated with caffeine at concentrations of (0–500 µM). Cell migration away from tumor spheroids was randomly captured at 0, 3, 5, and 7 days by an inverted microscope (Olympus, Melville, NY, USA). The tumor cell migration area was measured using the ImageJ program (National Institutes of Health, Bethesda, MD, USA).

### 4.12. Western Blot Analysis

The cells were cultured in 6-well plates at a density of 5 × 10^5^ cells/well overnight and then treated with different concentrations of caffeine (0–500 µM) for 24 h. The treated cells were washed with ice-cold PBS and incubated with RIPA lysis buffer containing commercial protease inhibitor (Cell Signaling Technology, Danvers, MA, USA) on ice for 45 min. Next, the cell lysates were harvested by scraping and centrifuged at 12,000 rpm for 5 min. The protein contents of the obtained supernatants were determined with a bicinchoninic acid (BCA) protein assay kit (Thermo Fisher Scientific, Rockford, IL, USA) according to the manufacturer’s protocol. After heating at 95 °C for 5 min, equal amounts of protein samples (50–60 µg) were separated by sodium dodecyl sulfate polyacrylamide gel electrophoresis (SDS-PAGE) and transferred to polyvinylidene difluoride (PVDF) or nitrocellulose membrane (Bio-Rad, Hercules, CA, USA). Next, the membranes were blocked for 1 h at room temperature in 5% (*w/v*) non-fat dry milk in TBS-T (Tris-buffer saline: 25 mM Tris-HCl and 125 mM NaCl, and 0.1% tween 20) and then incubated with the appropriate primary antibodies at 4 °C overnight. Then, the membranes stained with secondary antibodies for 2 h at room temperature. Finally, the bands of protein expression levels were detected by Super Signal West Pico chemiluminescent substrate (Pierce, Rockford, IL, USA). The intensity of the protein band was quantified using ImageJ software (National Institutes of Health, Bethesda, MD, USA) and normalized with β-actin.

### 4.13. Immunofluorescence Assay

After treatment with caffeine at different concentrations for 24 h, the treated cells were fixed with 4% (*v/v*) paraformaldehyde for 15 min and permeabilized with 0.1% (*v/v*) Triton X-100 for 10 min at room temperature. Next, the cells were incubated with blocking buffer (10% (*v/v*) FBS in PBS containing 0.1% (*v/v*) Triton X-100) at room temperature for 1 h and then incubated with a recommended dilution of primary antibodies (integrin αv, integrin β3, and c-Myc) in blocking buffer at 4 °C overnight. After washing threes time with PBS, the cells were co-stained with Alexa Fluor 488 or Alexa Fluor 594-conjugated goat anti-rabbit IgG (1:250 ratio) and Hoechst 33342 (10 µg/mL) in blocking buffer for 1 h at room temperature in the darkness. Lastly, the stained cells were covered with 50% (*v/v*) glycerol in PBS. Confocal images were captured under fluorescence microscopy (Nikon ECLIPSE Ts2, Tokyo, Japan) and the fluorescence intensity was measured by ImageJ software (National Institutes of Health, Bethesda, MD, USA).

### 4.14. Statistical Analysis

All results are representative of at least three independent experiments. The data were presented as the mean ± standard deviation (SD). Statistical differences between the means were investigated by one-way analysis of variance (ANOVA) followed by Fisher’s least significant difference (LSD) test at a significance level of *p* < 0.05. SPSS statistics version 19 was used to analyze all data.

## 5. Conclusions

**Figure 7:**
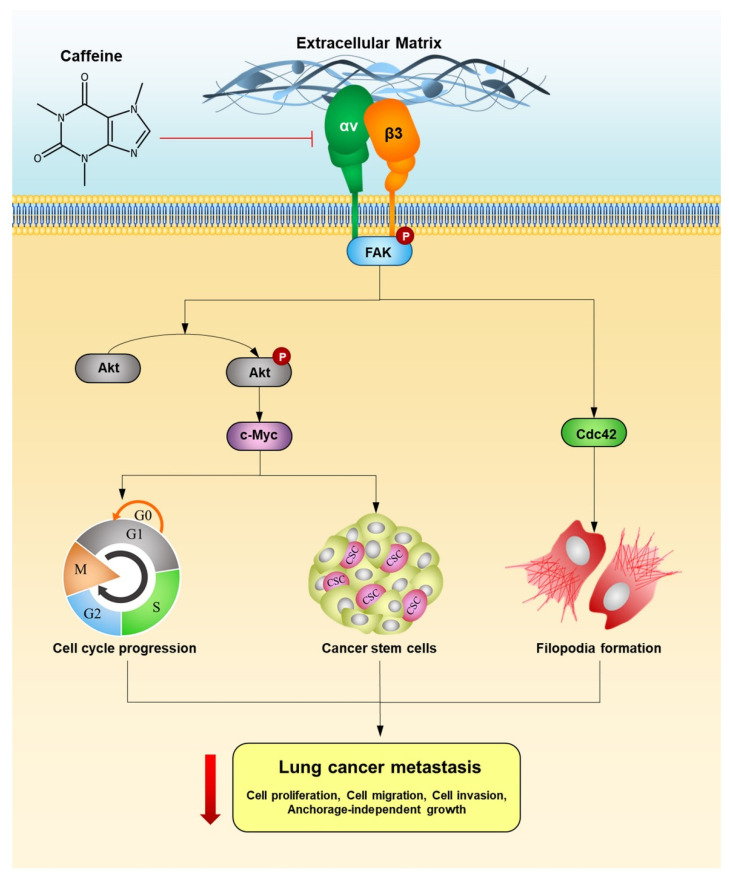
Schematic overview of the effect and mechanism of caffeine on inhibiting metastasis-related behaviors in human lung cancer cells.

The present study revealed that caffeine inhibits the proliferation of human lung cancer NCI-H23 cells by enhancing G1/G0 cell cycle arrest. In term of metastasis, caffeine could suppress the ability of cancer cells to migrate, invade, and grow in an anchorage-independent manner. Moreover, caffeine has the potential to attenuate the formation of tumor spheroids of lung CSC-enriched populations. The mechanisms supporting the action of caffeine associated with metastasis-related behaviors, involve the suppression of the integrin αv, β3, and FAK/Akt/c-Myc signaling pathway. A schematic overview of the effect and mechanism of caffeine-inhibiting metastasis-related behaviors in human lung cancer cells is presented in Figure 7. Our research findings show the potentially beneficial effect of caffeine in the prevention of human lung cancer metastasis.

## Data Availability

Data is contained within the article.

## Sample Availability

Samples of the compounds are not available from the authors.
