# Correction: Meisaprow et al. Caffeine Induces G0/G1 Cell Cycle Arrest and Inhibits Migration through Integrin αv, β3, and FAK/Akt/c-Myc Signaling Pathway. *Molecules* 2021, *26*, 7659

**DOI:** 10.3390/molecules29112642

**Published:** 2024-06-04

**Authors:** Pichitchai Meisaprow, Nithikoon Aksorn, Chanida Vinayanuwattikun, Pithi Chanvorachote, Monruedee Sukprasansap

**Affiliations:** 1 Graduate Student in Master of Science Program in Nutrition, Faculty of Medicine Ramathibodi Hospital and Institute of Nutrition, Mahidol University, Bangkok 10400, Thailand; pichitchai.mei@student.mahidol.ac.th; 2 Department of Clinical Pathology, Faculty of Medicine Vajira Hospital, Navamindradhiraj University, Bangkok 10300, Thailand; nithikoon@nmu.ac.th; 3 Division of Medical Oncology, Department of Medicine, Faculty of Medicine, Chulalongkorn University, Bangkok 10330, Thailand; chanida.vi@chula.ac.th; 4 Cell-Based Drug and Health Product Development Research Unit, Faculty of Pharmaceutical Sciences, Chulalongkorn University, Bangkok 10330, Thailand; 5 Department of Pharmacology and Physiology, Faculty of Pharmaceutical Sciences, Chulalongkorn University, Bangkok 10330, Thailand; 6 Food Toxicology Unit, Institute of Nutrition, Mahidol University, Salaya Campus, Nakhon Pathom 73170, Thailand

In the original publication [1], there were mistakes in Figures 1c,g and 3c as published. The same sets of Hoechst/PI staining result images at a caffeine concentration of 250 µM were unintentionally provided for two different time groups in Figure 1c,g. The image of the cell invasion assay at a caffeine concentration of 100 µM was mistakenly shown alongside the results of the control group in Figure 3c. The corrected Figure 1c,g and Figure 3c appear below. The authors state that the scientific conclusions are unaffected. This correction was approved by the Academic Editor. The original publication has also been updated.

**Figure 1:**
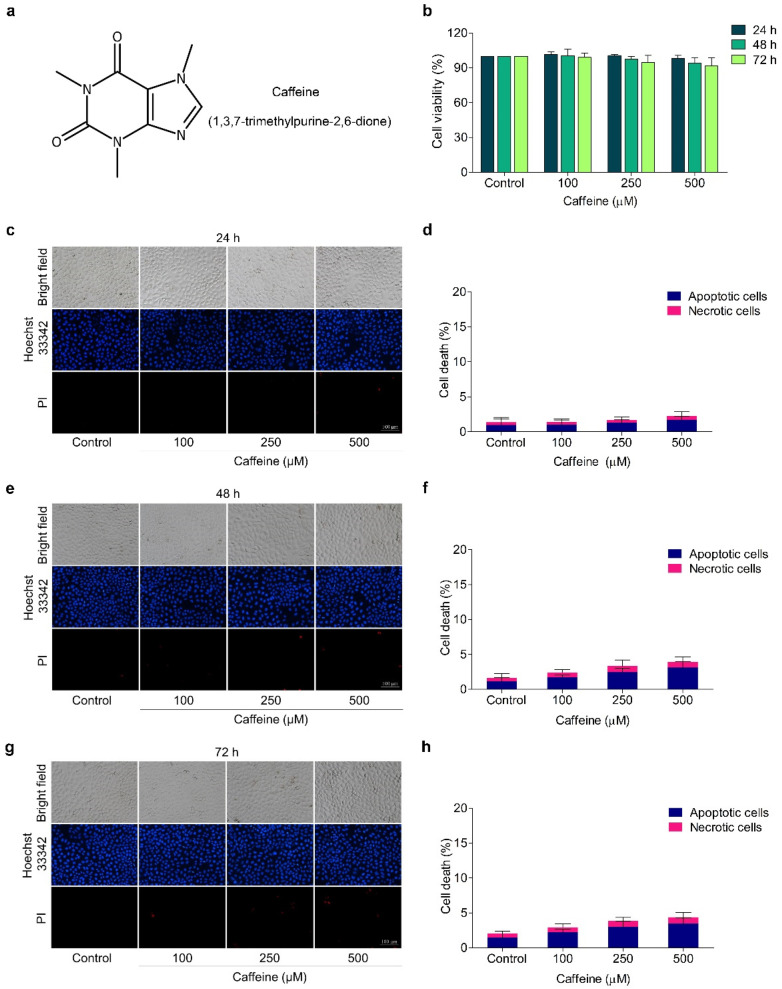
Effect of caffeine on viability of NCI-H23 cells. (**a**) Chemical structure of caffeine. (**b**) The cells were treated with caffeine at various concentrations (0–500 µM) for 24–72 h and cell viability was investigated with MTT assay. (**c**–**h**) The cells were treated with 0–500 µM of caffeine for 24–72 h and apoptotic and necrotic cells were examined by co-staining with Hoechst 33342 and PI. Images were visualized by fluorescence microscopy. Data are shown as mean ± SD (*n* = 3).

**Figure 3:**
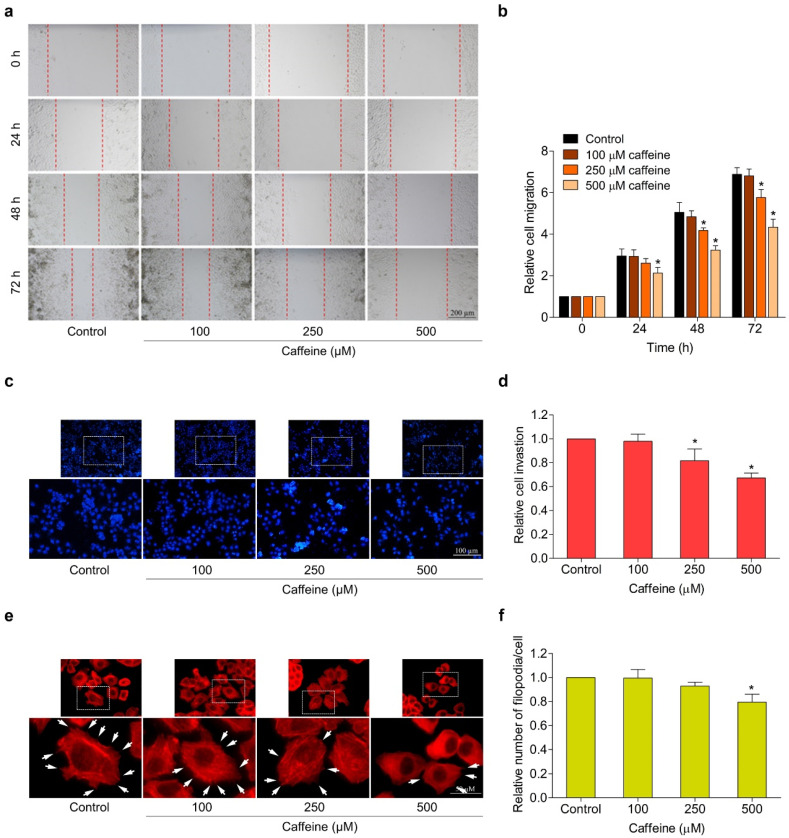
Effect of caffeine on cell migration, invasion, and filopodia formation in NCI-H23 cells. (**a**) Cell migration activity was assessed by wound-healing assay. The cells were treated with non-toxic doses of caffeine, and the movement of cells into the wound space was evaluated at 0, 24, 48, and 72 h. (**b**) The cell migration rate was represented as a relative value. (**c**) Cell invasion was determined with the transwell Boyden chamber. After incubating with caffeine for 24 h, the invaded cells were stained with Hoechst 33342 and determined by fluorescence microscopy. (**d**) Relative cell invasion was calculated from the number of invaded cells in the treatment groups divided by the control group. (**e**) The cells were cultured with caffeine for 24 h before staining with phalloidin–rhodamine. Filopodia formation was visualized by fluorescent microscopy and is indicated by white arrows. (**f**) The number of filopodia per cell was calculated as a relative value. Data are presented as mean ± SD (*n* = 3). * *p* < 0.05 compared with the non-treated control.
